# REPLY BY THE AUTHORS: Re: An unanswered question in pediatric urology: the post pubertal persistence of prepubertal congenital penile curvature correction by tunical plication

**DOI:** 10.1590/S1677-5538.IBJU.2017.0055.2

**Published:** 2018

**Authors:** Unsal Ozkuvanci, Orhan Ziylan, M. Irfan Donmez, Omer Baris Yucel, Tayfun Oktar, Haluk Ander, Ismet Nane

**Affiliations:** 1 Department of Urology, Istanbul Faculty of Medicine, Istanbul University, Istanbul, Turkey

*To the editor*,

We appreciate Prof. Yachia's interest in our article (1). There are two main questions with regard to the treatment of congenital penile curvature (CPC) in pediatric age group. Those are timing and technique.

Previously, post pubertal correction was more commonly performed with the belief of the ongoing developmental process of the penis and some also authors believed the spontaneous correction of the curvature with time. Most probably, reason for this approach may be attributed to the relatively late recognition of CPC in children. Moreover, the use of adult techniques in pediatric population (shortening and lengthening etc.) and lack of long-term surgical results have prepared the grounds for discussion.

After unveiling penile neuroanatomy and the definition of dorsal midline plication technique by Baskin et al. (2), it has become globally popular within pediatric urologists in the correction of mild to moderate penile curvature with or without hypospadias. However, recurrence of curvature has been observed in some patients with hypospadias. In addition, there have been very few pediatric reports regarding the long-term results of patients with CPC in terms of recurrence.

In Prof. Yachia's article, it has been postulated that plication only method may not provide the fibrosis that has been made with incision or excision. However, there has been no long-term (post pubertal outcome of pre pubertal surgery) data about shortening excisional/incisional methods or lengthening methods. Thus, this idea has not been supported with evidence-based literature.

Our practice was correcting mild-moderate curvature during circumcision. When we analyzed our results, we came across with 8/13 recurrence rate in the long-term (mean 7 years of follow-up). As Prof. Yachia has proposed the term post pubertal persistence can be replaced with ‘late onset recurrence’.

We still believe that technique is not solely responsible for recurrences since there have been no comparative studies involving both techniques. In our cohort, even more severe curvature (when compared with pre operative degree) was observed in some patients. This finding can be attributed to the ongoing process of corporeal disproportion. Therefore, late onset recurrence of congenital penile curvature may depend on the developmental process as well as technical problems.
